# Biomechanical behavior of two different surface treatments on dental implants with healing chambers in osteoporotic rabbits: an experimental study

**DOI:** 10.1038/s41598-025-31217-5

**Published:** 2025-12-04

**Authors:** Sérgio Alexandre Gehrke, Antonio Scarano, Felice Lorusso, Jaime Aramburú Júnior, Tiago Luis Eilers Treichel, Artiom Lijnev, José Eduardo Maté Sánchez de Val, Eleani Maria da Costa

**Affiliations:** 1 Department of Implantology, Bioface/Postgrados en Odontología, Universidad Catolica de Murcia, Montevideo, 11100 Uruguay; 2 Department of Biotechnology, Universidad Católica de Murcia, 30107 Murcia, Spain; 3 Department of Innovative Technologies in Medicine and Dentistry, University of Chieti-Pescara, 66013 Chieti, Italy; 4 Department of Physiology and Pharmacology, Pro-Rectorate of Graduate Studies and Research (PRPGP) of the Universidade Federal de Santa Maria, Santa Maria, 97105-900 Brazil; 5 Department of Surgery, Faculty of Medicine Veterinary, University of Rio Verde, Rio Verde, 75901-970 Brazil; 6 Department of Biomaterials Engineering, Faculty of Health Sciences, Universidad Católica de Murcia, 30107 Murcia, Spain; 7 Department of Materials Engineering, Pontificial Catholic University of Rio Grande do Sul, Porto Alegre, 90619-900 Brazil

**Keywords:** Dental implants, Osteoporosis, Histomorphometry, Implant stability, Implant surface treatment, Rabbit animal study, Bone–implant contact, Bone area fraction occupancy, Implant removal torque, Diseases, Medical research

## Abstract

The aging global population is experiencing a growing prevalence of metabolic bone diseases, particularly osteoporosis, which compromises bone quality and poses challenges for dental implant osseointegration. Despite the systemic bone fragility associated with osteoporosis, the use of implants is not contraindicated, although it may be accompanied by increased risks. Recent advances in implant macrogeometry and surface treatment aim to enhance osseointegration in compromised bone conditions. This study aimed to biomechanically and histologically evaluate the performance of dental implants featuring healing chambers and two different surface treatments in an animal model with induced osteoporosis. Twenty female New Zealand white rabbits were used, with osteoporosis induced via ovariectomy and glucocorticoid administration. A total of 80 titanium implants (two surface types: Group A – titanium oxide-blasted; Group B – titanium oxide-blasted plus HCl-conditioned) were installed in both rabbit tibiae (*n* = 2 implant per tibia). Stability was measured by resonance frequency analysis (RFA) and maximum removal torque. Histological assessments included bone-to-implant contact (BIC%) and bone area fraction occupancy (BAFO%) at 14- and 28-days post-implantation. RFA revealed increased implant stability over time in both groups, with Group B showing significantly higher ISQ values in 28 days (*p* < 0.0001). Removal torque values also improved over time, with Group B showing significantly greater values at 28 days (30.1 ± 4.18 Ncm) compared to Group A (25.6 ± 3.95 Ncm). Histomorphometric analysis showed that BAFO% was significantly greater in Group B at 28 days, but no significant differences in BIC% were observed between groups at either time point. Within the limitations of this animal model, implants with acid-etched surfaces showed improved biomechanical stability and bone occupancy at later healing stages, but only at 28 days. These results suggest that surface modifications may play a role in enhancing osseointegration over time in compromised bone environments. However, the findings are limited to this preclinical model and do not allow direct clinical extrapolation.

## Introduction

The growth of the world’s elderly population is evident, as life expectancy in developed and developing countries has increased considerably in the last decade and the trend is for an even greater increase in the coming years^1^. In this context, there is a change in the epidemiological profile of the population, with a significant increase in chronic and multiple diseases. Diseases of the circulatory system, neoplasms and metabolic bone diseases stand out in the scenario of diseases related to aging^2^. Osteoporosis is a systemic skeletal disorder characterized by reduced bone mass and microarchitectural deterioration, which may be influenced by chronic low-grade inflammation. It is a multifactorial disease commonly associated with calcium deficiency, glucocorticoid therapy, physical inactivity, or impaired calcium absorption^3,4^. Furthermore, osteoporosis has been described as a multifactorial disease that weakens bones due to the body’s poor calcium absorption. This results in low bone mineral density, which disorganizes the bone’s microarchitecture and leads to fractures that compromise the well-being of the elderly, with a high incidence in old age^5^.

Titanium endosseous dental implants have been successfully used for functional and aesthetic rehabilitation with predictable outcomes, provided there is adequate bone quality and quantity^6^. However, these conditions are not always present, especially in elderly patients, due to systemic changes in sex hormone levels in postmenopausal women. When estrogen production ceases or is drastically reduced, it leads to significant bone mass loss^7^. As a result, osteoporosis develops, characterized by an imbalance where osteoclast activity exceeds osteoblast activity. Under normal health conditions, bone resorption and formation are balanced, a process known as bone turnover. However, in osteoporosis, this balance is disrupted, leading to greater bone resorption than new formation.

On the other hand, therapy with osseointegrated implants is not contraindicated for patients with osteoporosis, however, they may be more prone to risks of complications^8,9^. Both systematic reviews cited^8,9^ showed that there was no significant difference in the survival rate of dental implants installed in patients with osteoporosis when compared to patients without osteoporosis, unlike in the item of peri-implant bone loss, which revealed a greater difference in peri-implant bone tissue loss for patients with osteoporosis. This bone loss reduces the bone-implant contact area, which can be one of the complications of long-term dental implant therapy associated with osteoporosis^10^.

Several studies on new implant designs and new surface treatments have been developed and presented in the literature as beneficial for osseointegration, potentially promoting an improvement and/or acceleration in this process^11–14^. In this sense, an implant model was developed with a new macrogeometry featuring healing chambers, which seek to generate less bone compression during implant installation, thus benefiting the osseointegration process^11,12^. Furthermore, this implant model with healing chambers recently received a new surface treatment, which was also developed with the aim of benefiting the healing of the bone tissue around the implants^13^. However, most of these studies are developed under ideal conditions, that is, in organisms (animals or humans) that do not present systemic alterations that could compromise the results.

Thus, the present study was developed to biomechanically evaluate the behavior of implants with healing chambers and two different surfaces in animals with induced osteoporosis. To assess stability, resonance frequency analysis (RFA) and maximum removal torque force were used. Histological and histomorphometric analyses were performed to evaluate the bone characteristics around the implants. These evaluations were conducted during the initial period of osseointegration, specifically after 14 and 28 days of implant installation.

## Materials and methods

The present study was divided into two phases. The first was the induction of osteoporosis, which was achieved by the waiting time between induction and the second stage of implant installation, which was seven months. The second phase consisted of surgeries to install the implants in these animals and the analysis of osseointegration. The present study was reported in accordance with ARRIVE guidelines (https://arriveguidelines.org). Furthermore, the ethical principles of the National Council for the Control of Animal Experimentation (CONCEA) were followed, as well as the concern for animal welfare in accordance with Law No. 11,794 of October 8, 2008 (Procedures for the Scientific Use of Animals).

### Animal model and management

Firstly, this study was submitted and approved by the Animal Use Ethics Committee (CEUA) of the University of Rio Verde under number 06/2020 (on 04/21/2021). Twenty female New Zealand white rabbits (Oryctolagus cuniculus) from the vivarium of the University of Rio Verde (UnRV, Rio Verde, Brazil), aged six months and with an average initial body mass of 3 kg, were used. Rabbits were used for this study, as it is an animal model consolidated in the literature for studying dental implants^14,15^. The animals were housed individually in cages suitable for rabbits (size 50 cm x 50 cm x 50 cm x 50 cm), with drawers for collecting feces and urine, individual drinkers and feeders as recommended by the National Council for the Control of Animal Experimentation (CONCEA) in its resolution number 15 of 2013. The animals were housed prior to execution for a minimum period of 15 days, for their adaptation to environmental conditions and human coexistence, as well as for detection of possible diseases. For this purpose, they were fed with industrialized feed for this animal model and with water ad libitum. These conditions will be maintained throughout the study execution period.

### Anesthetic procedure

Pre-anesthetic medication consisted of a combination of ketamine hydrochloride at 20 mg/kg (Vetnil, Louveira, Brazil), midazolam maleate at 2 mg/kg (Dormire^®^ - Cristália Produtos Químicos Farmacêuticos, São Paulo, Brazil) and morphine sulfate at 3 mg/kg (Dimorf - Cristália Produtos Químicos Farmacêuticos, São Paulo, Brazil), all administered intramuscularly (IM). Anesthetic induction and maintenance were performed with Isofluorane (Syntec, Tamboré, Brazil) to effect, using a mask, with an open inhalation system, vaporized in 100% oxygen, with spontaneous breathing.

### Osteoporosis induction procedure

The animal model for inducing osteoporosis was adapted from the study by Permuy et al.^14^, which evaluated various methods for inducing osteoporosis and concluded that a combination of ovariectomy and glucocorticoid administration is simple, reliable, and reproducible. This approach leads to observable osteoporotic bone changes within just 6 weeks. The method involves performing ovariectomy first, followed by the administration of glucocorticoids.

For the bilateral ovariectomy surgical procedure, the Snook hook technique was employed. First, the abdominal region was shaved, and antisepsis was carried out using a topical 2% Chlorhexidine Gluconate solution. A 2.0 cm incision was then made in the retro-umbilical area, and the ovaries were located and exposed with the aid of a Snook hook. Next, a Kelly clamp was used to secure the ovarian pedicle, mesovarium, mesosalpinx, and part of the uterine tube. A second clamp was placed 5 mm above the first. A 2 − 0 nylon suture was then used to ligate the ventral ovarian pedicle below the upper clamp, and the ovaries were excised above this clamp. Afterward, the remaining clamp was removed, and the muscles were closed with standard Sultan sutures using 2 − 0 nylon. The skin was closed with the same suture material using simple interrupted stitches. Postoperative care was provided as outlined below.

Finally, the induction of osteoporosis was complemented by the administration of Methylprednisolone Sodium Succinate (Meticorten, Merck & Co. Inc., Rahway, USA) at a dose of 1 mg/kg/day via IM for four weeks according to the protocol described by Castañeda et al.^16^. However, the animals were kept for seven more months for the second stage of implant installation.

### Implant characteristics and group formation

**Fig. 1:**
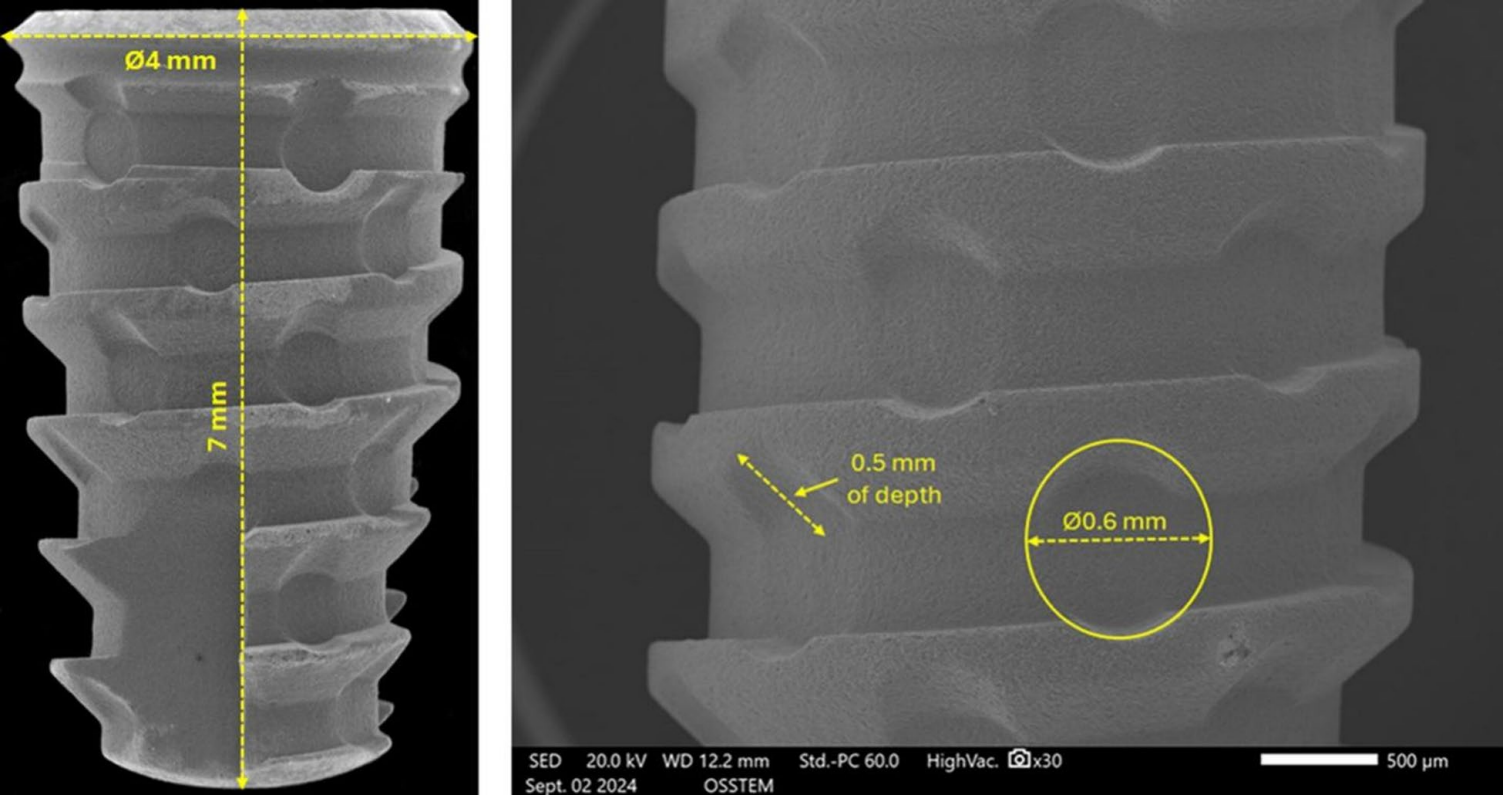
Scanning electron microscopy (SEM) images of the implants used in the study. (Left) General view of the implant showing its macrogeometry: conical design with healing chambers, 4 mm in diameter and 7 mm in height. (Right) Detail of a healing chamber, showing 0.5 mm of depth and 0.6 mm of diameter.

A total of 80 screw dental implants manufactured in grade IV titanium (Maestro implant, Implacil/Osstem, São Paulo, Brazil) were used in the present study. All implants had the same macrogeometry (conical with healing chambers) and dimensions (diameter and heigth), as shown in Fig. 1.

**Fig. 2:**
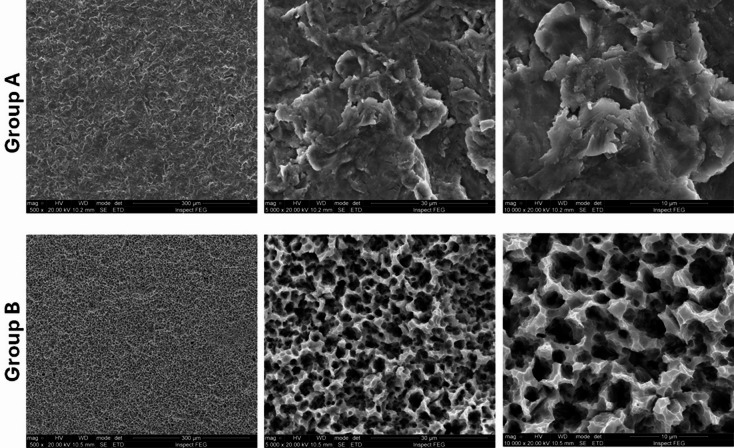
Scanning electron microscopy (SEM) images showing surface topography of the implants in Group A (upper row) and Group B (lower row) under different magnifications (500×, 5000×, and 10,000×). Group A implants were treated by sandblasting with titanium oxide microparticles. Group B implants were sandblasted with titanium oxide microparticles and subsequently etched with 35% hydrochloric acid (Superiore surface).

Two distinct surface treatments were tested, forming the experimental groups (*n* = 40 implants per group): **group A**, where the implants had a surface blasted with titanium oxide microparticles; **group B**, where the implants were blasted with titanium oxide microparticles and subsequently received conditioning with hydrochloric acid (HCl) at a concentration of 35% (Superiore surface). Each group (A and B) was further subdivided based on healing time, with 20 implants evaluated at 14 days and 20 implants at 28 days, totaling 80 implants in the study. Figure 2 shows SEM images with different magnifications of both groups.

### Implant surgical procedures

At the time of surgery to install the implants, the animals weighed an average of 4.5 kg. Animals under general anesthesia underwent trichotomy in the tibia region and topical application of 1% Polyvinyl-Pyrrolidone Iodine solution. Furthermore, both tibias received local anesthesia by infiltration of Mepivacaine 2% with Epinephrine (DFL, Rio de Janeiro, Brazil). A 4 cm linear incision was made on the medial side of the hind paw, the tissues were dissected in layers until reaching the bone tissue, then the bone bed was drilled with the drill sequence recommended by the manufacturer for this implant model under abundant cooling with saline solution. After the implants were screwed into the bone using the appropriate driver, always with the same distribution in all animals: in the right tibia, implant of group A in the proximal position and implant of group B in the most distal position; in the left tibia the sequence was reversed. Finally, the suture was made using 4.0 nylon thread with simple isolated stitches.

### Post-operative care and euthanasia

All animals received post-operative care in accordance with the principles of the 3Rs (Replacement, Reduction, and Refinement) and following the ethical guidelines established by the Brazilian National Council for the Control of Animal Experimentation (CONCEA) and international standards for animal research^17^. Special attention was given to minimizing animal pain and distress throughout the study.

After implant placement, animals were monitored daily by a veterinarian for signs of pain, infection, behavioral changes, or any complications. The following medications were administered subcutaneously as part of the post-operative protocol:

- Meloxicam (Meloxiflex, MundoAnimal, Pindamonhangaba, Brazil) – 0.2 mg/kg once daily for 3 days (anti-inflammatory);
- Tramadol hydrochloride (Cronidor, União Química, São Paulo, Brazil) – 5 mg/kg three times daily for 3 days (analgesic);
- Penicillin G-benzathine (Penfort, Ourofino, Cravinhos, Brazil) – 80,000 IU/kg once every 3 days, for two doses (antibiotic prophylaxis).

Throughout the study period, no unexpected deaths or adverse events were recorded, and no animals required replacement. The sample size was determined a priori and maintained throughout the experiment, ensuring adherence to the principle of Reduction by avoiding unnecessary animal use while maintaining adequate statistical power.

Euthanasia was performed at predetermined time points: 14 days (*n* = 5 animals) and 28 days (*n* = 5 animals) post-implantation. The method followed CONCEA’s guidelines (Normative Resolution No. 37/2018)^18^, using an intravenous overdose of 3% Pentobarbital (Euthanyle, São Paulo, Brazil) at a dose of 50 mg/kg. Death was confirmed by the attending veterinarian through the absence of heartbeat, respiratory movement, pulse, corneal reflex, and the presence of pale mucous membranes.

All procedures in this study were designed and conducted to minimize animal suffering and maximize the quality of scientific outcomes, in strict compliance with ethical and legal requirements.

### Stability measurement by resonance frequency analysis (RFA) and maximum removal torque

The stability (E) of each implant was measured immediately after installation (E1) and in the samples after collection at each predetermined sacrifice time, that is, 14 days (E2) and 28 days (E3). For this purpose, a magnetic sensor (SmartPeg) number 26 was screwed onto the implant and, with the Osstell^®^ device (Osstell AB, Gothenburg, Sweden), the implant stability quotient (ISQ) was measured in two directions for each sample: proximodistal (PD) and lateral-medial (LM). Immediately after the ISQ measurement, half of the samples from each group at each time point (*n* = 10 per group/time) were removed from the bone and the maximum removal torque was measured. This procedure was performed on computerized equipment to measure torque (CME-30, Osvaldo Filizola, São Paulo, Brazil). Additionally, images of the implants after removal were taken by scanning electron microscopy to visually assess the amount of bone remaining in these samples.

### Sample collection, treatment and histological analysis

The samples intended for histological analysis (*n* = 10 per group/time), after collection, were immediately immersed in 10% buffered formalin and kept in this solution for 7 days. They were then dehydrated in a sequence of ethanol solution (50–100%) and embedded in historesin (Technovit 7200 VLC, Kulzer, Wehrheim, Germany). Sections were performed using an IsoMet 1000 machine (Buehler, Lake Bluff, IL, USA). A slide of the central part of each implant was obtained from each sample. The slides were stained using the hematoxylin-eosin technique. Histomorphometry was performed using a light microscope (E200, Nikon, Tokyo, Japan). For histometric measurements, the ImageTool for Microsoft Windows software (version 5.02, University of Texas Health Science Center, San Antonio, CA, USA) was used.

### Analysis used to confirm osteoporosis in animals

To confirm osteoporosis in the animals, digital periapical radiographs were taken of the most proximal portion of the tibia, in regions where implants were not installed. These bone portions correspond to the remaining ends of the blocks after the cuts made to include the samples in resin.

As a control for the osteoporosis induction process, ten tibia samples from remaining blocks of healthy rabbits were used, previously used in another study conducted by our research group, in which the animals presented similar characteristics (lineage, age, weight and experimental conditions) to those of the present study. This study was published by our research group^13^.

### Bone density analysis

Radiographic images of the proximal region of the rabbit tibiae were acquired after resin embedding using a portable digital radiography system (IriX-ray DX 3000; Dexcowin, Seoul, Korea), positioned at a fixed distance of 15 cm from the sample. Each bone block was placed directly on an intraoral digital sensor (RVG First, Trophy, Toulouse, France), and image acquisition was performed using Trophy Imaging software (version 7.0.19.3.d1). For quantitative analysis, the images were processed using ImageJ software (version 1.52v; National Institutes of Health, Bethesda, MD, USA). All radiographs were first converted to 8-bit grayscale and then binarized by adjusting the threshold to isolate mineralized tissue, which appeared black in the binary images.

**Fig. 3:**
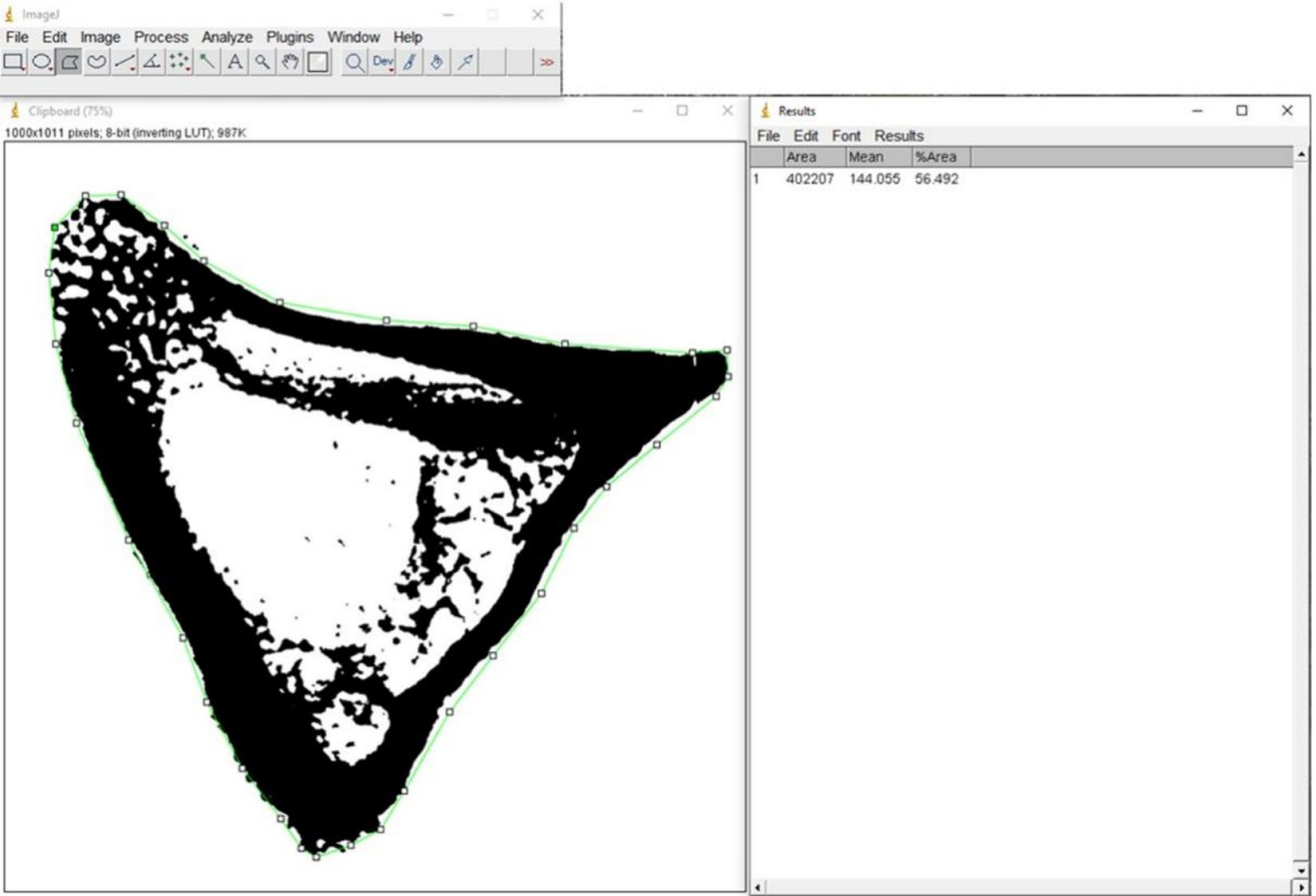
Representative binarized radiographic image of the proximal tibial epiphysis of a rabbit. The region of interest (ROI) was manually delineated (green outline), and the black area represents mineralized bone tissue. Image analysis was performed using ImageJ software to calculate the percentage of the area occupied by bone tissue (%Area), used as an indicator of apparent bone density.

The region of interest (ROI), corresponding to the bone area of the proximal tibial epiphysis, was manually delineated using the polygon selection tool. After segmentation, quantitative analysis was performed using the *“Analyze > Measure”* function, which provided the following parameters: total area of the ROI (pixels²); area occupied by bone tissue (pixels^2^); mean gray value; percentage of area occupied by bone tissue (%Area). The percentage of area occupied by bone tissue was used as an indicator of the apparent bone density in the analyzed region. Figure 3 shows a binarized radiographic image along with the manually delineated region of interest (ROI) and the corresponding measurement of the black area.

### Bone-implant contact percentage (BIC%) and percentage of bone area fraction occupancy (BAFO%) measurements

**Fig. 4:**
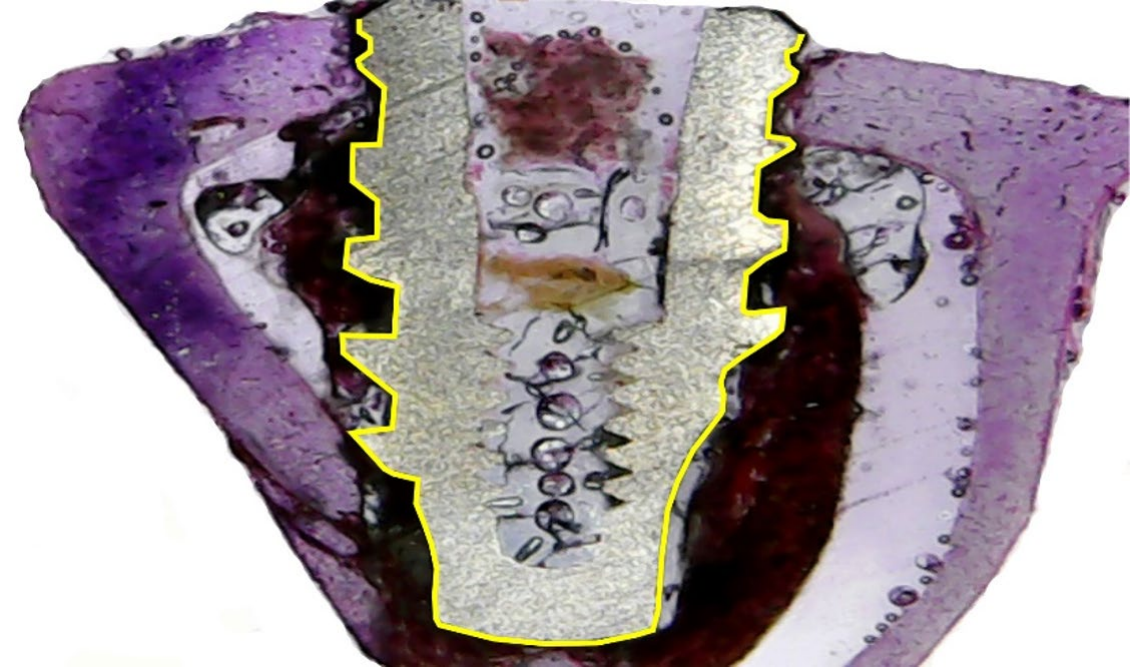
Image showing the bone-implant contact percentage (BIC%) measured around each implant sample (yellow line).

The bone-implant contact percentage (BIC%) was defined as the amount of bone tissue in contact with the titanium surface. Measurements were made across the entire length of the implant (Fig. 4).

**Fig. 5:**
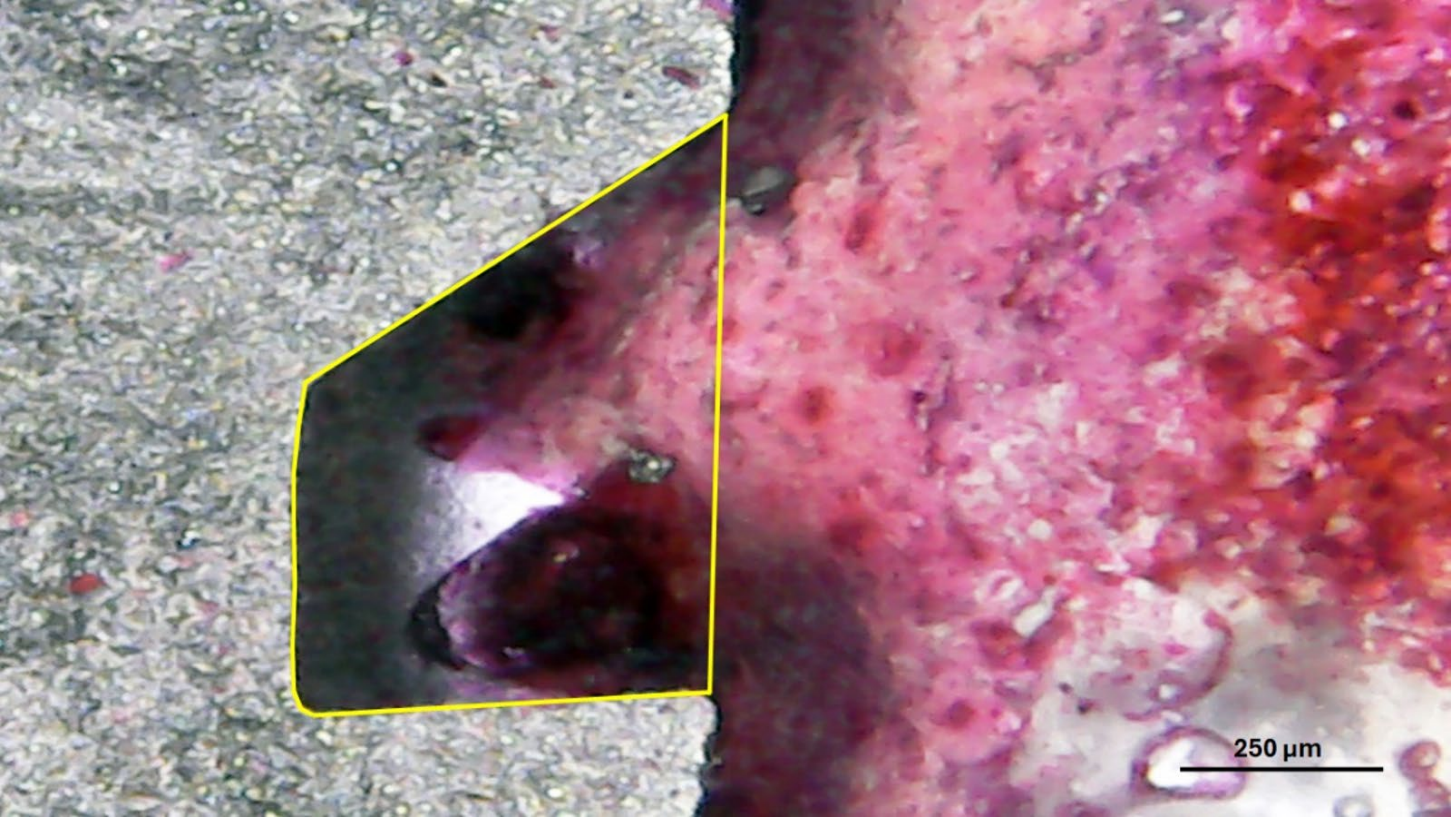
Representative image of a thread where the bone area fraction occupancy percentage (BAFO%) was measured (inside of the yellow lines).

The percentage of bone area fraction occupancy (BAFO%) was defined as the fraction of occupied bone tissue within the area of ​​each thread in the histological section of the right side of each implant (Fig. 5). All threads were measured and included in the statistical analysis.

### Statistical analysis

All quantitative data obtained from the RFA (ISQ values), maximum removal torque values, and histomorphometric parameters (BIC% and BAFO%) were analyzed using appropriate statistical tests based on data distribution and study design. Initially, data normality was assessed using the Shapiro-Wilk test. For comparisons between groups and time points, either two-way ANOVA or the Kruskal-Wallis test was applied, depending on whether assumptions of normality and homogeneity of variances were met. When ANOVA was used, Tukey’s post hoc test was conducted to identify specific differences between means. For non-parametric data, the Mann-Whitney U test was employed for pairwise comparisons. The Student’s t-test was used when comparing two independent groups with normally distributed data. Statistical analyses were performed using GraphPad Prism 5 (GraphPad Software Inc., San Diego, CA, USA), with a significance level set at α = 0.05.

Cohen’s d was used to calculate the effect size for the post hoc power analysis. Based on the mean and standard deviation values (effect size = 1.11, α = 0.05, *n* = 10 per group), the analysis indicated a statistical power of approximately 0.88 (88%), which is above the commonly accepted threshold of 80%, suggesting that the sample size was adequate for this study.

## Results

### Bone density

**Fig. 6:**
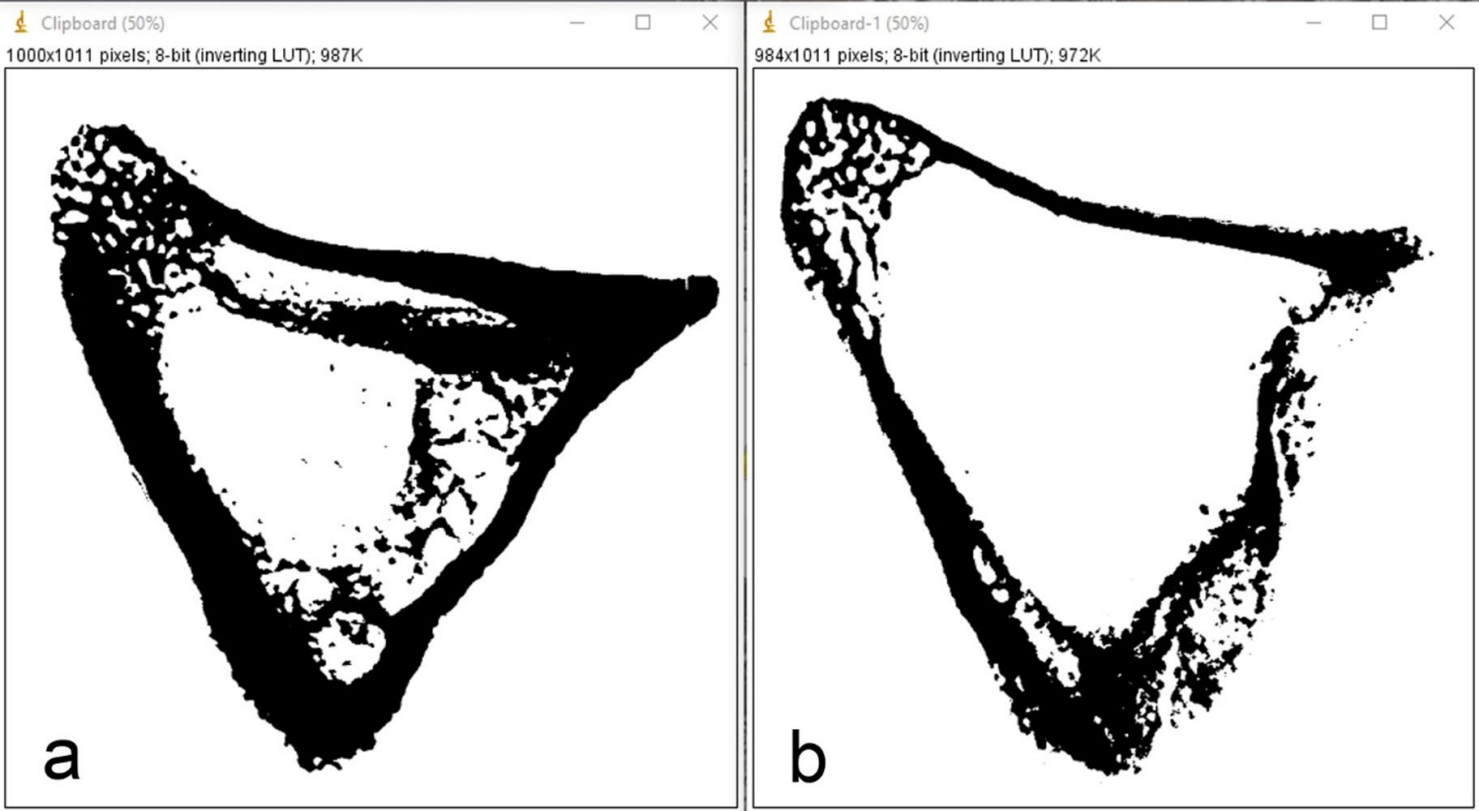
Binarized radiographic images of the proximal tibial epiphysis of rabbit tibiae. (**a**) Healthy control group; (**b**) induced osteoporosis group. Black areas represent mineralized bone tissue. A marked reduction in mineralized area is evident in the osteoporotic sample.

Radiographic analysis demonstrated a significant reduction in bone density in animals with induced osteoporosis compared to healthy controls. Figure 6 presents representative binarized radiographic images of the proximal tibial epiphysis from a control animal (Fig. 6a) and an osteoporotic animal (Fig. 6b), clearly showing the difference in the area occupied by mineralized tissue.

**Fig. 7:**
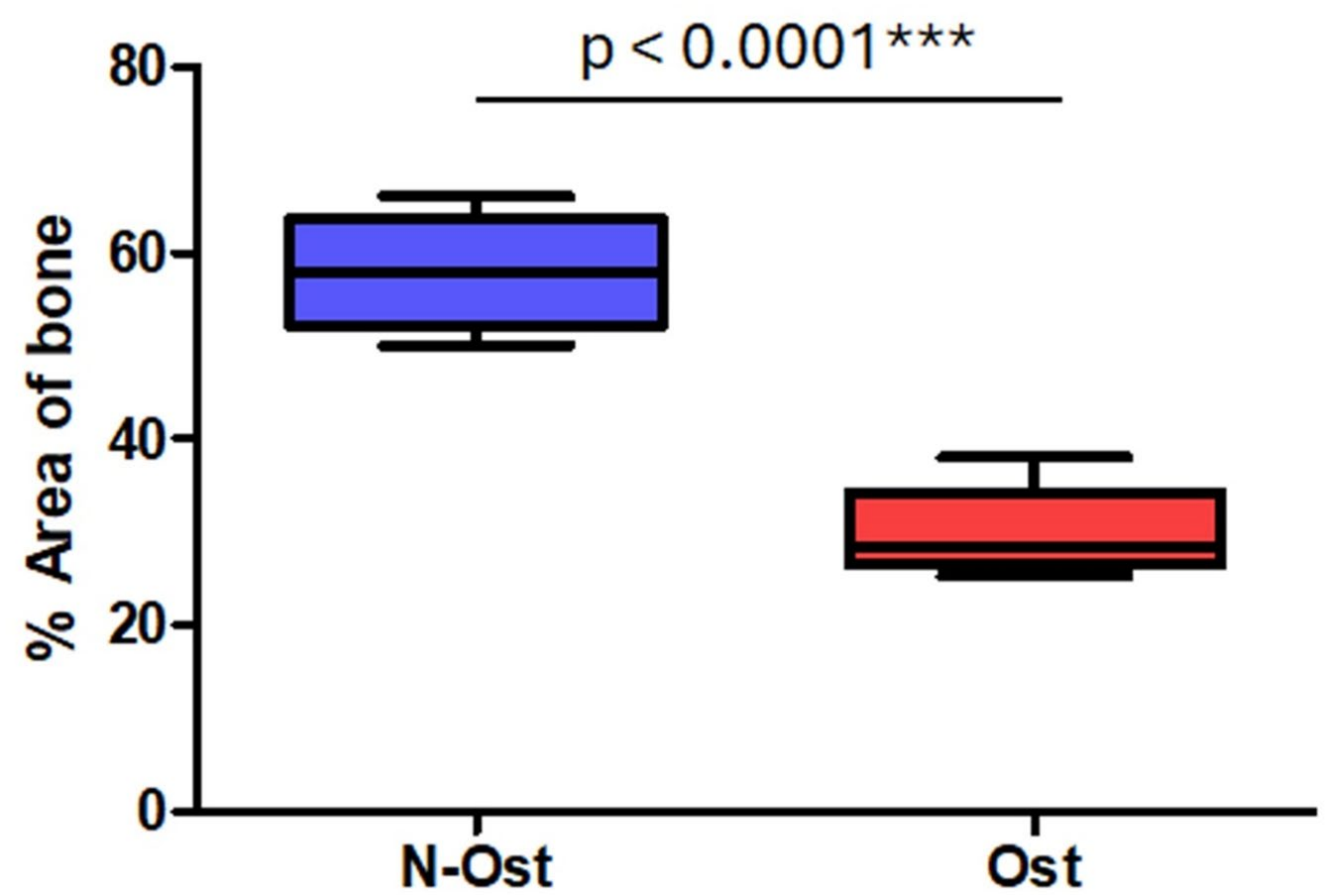
Box plot showing the distribution of bone density values (% mineralized area) in the proximal tibial epiphysis of healthy control animals and animals with induced osteoporosis. N-Ost = no osteoporosis; Ost = induced osteoporosis.

No statistically significant differences in bone density were observed between animals sacrificed at 14 and 28 days. Therefore, the values were pooled and presented as a single average per group for clarity. Quantitative analysis confirmed these findings: the healthy animals showed a mean mineralized area of 57.62 ± 5.77%, while the osteoporotic group exhibited a significantly lower value of 30.05 ± 4.18% (*p* < 0.0001) (Fig. 7). These results validate the successful induction of osteoporosis in the experimental model and provide an objective basis for interpreting the biological response of the implants under osteoporotic conditions.

### Stability measurement by resonance frequency analysis (RFA)

**Fig. 8:**
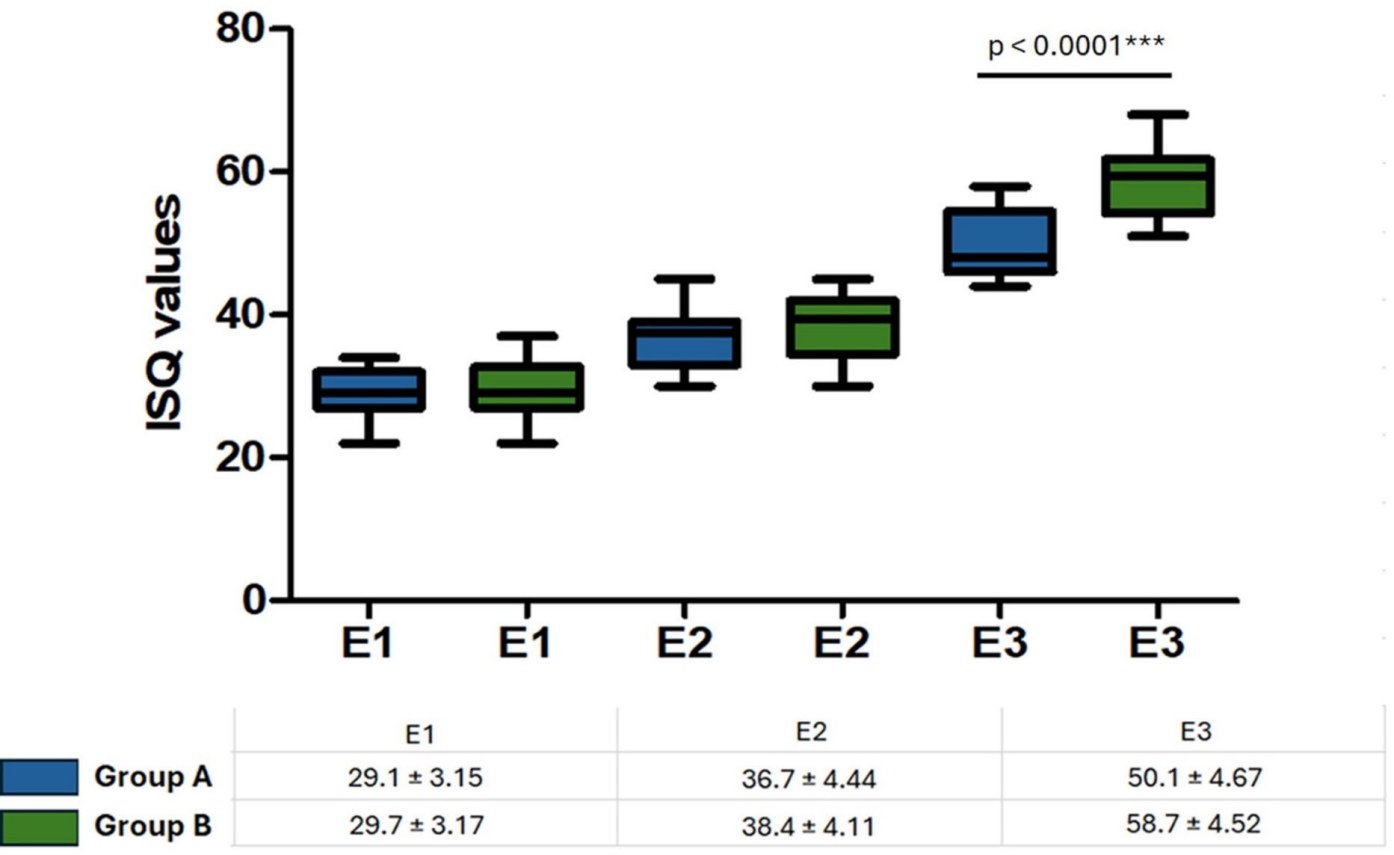
Box plot graph showing the evolution of ISQ in the 3 evaluated moments: E1 = moment of implant installation, E2 = 14 days later, and E3 = 28 days later. The table shows the mean values ​​and standard deviation for each group. Group A = implants with sandblasted titanium oxide surface; Group B = implants with sandblasted + hydrochloric acid-etched (35%) surface (Superiore surface).

The results obtained in the measurement of stability through RFA were organized into three different evaluation moments: E1, E2 and E3. Figure 8 shows in the line graph and table the evolution of stability for both groups in each of the time intervals evaluated.

Furthermore, the statistical analysis revealed significant differences between the groups for moment E3 (*p* < 0.0001), while at moments E1 and E2 they were not statistically significant (*p* = 0.6166 and 0.1790, respectively). These results indicate a trend of improvement in stability in both groups over time, with a more pronounced change in group B, especially after the third assessment moment. The significant p-value at E3 (28 days) suggests that at this stage the difference between groups becomes substantial, which may reflect differences in the treatments performed in each group.

### Results of implant removal torque values

The mean values ​​and standard deviation of implant removal torque were: 19.5 ± 4.20 Ncm for group A and 20.8 ± 4.34 Ncm for group B in 14 days. At 28 days after the implantation, the values ​​were 25.6 ± 3.95 Ncm (group A) and 30.1 ± 4.18 Ncm (group B). No significant difference was observed between the groups at 14 days, but a difference was found at 28 days. Statistical analysis showed significant differences between the evaluation times for samples from the same group.

**Fig. 9:**
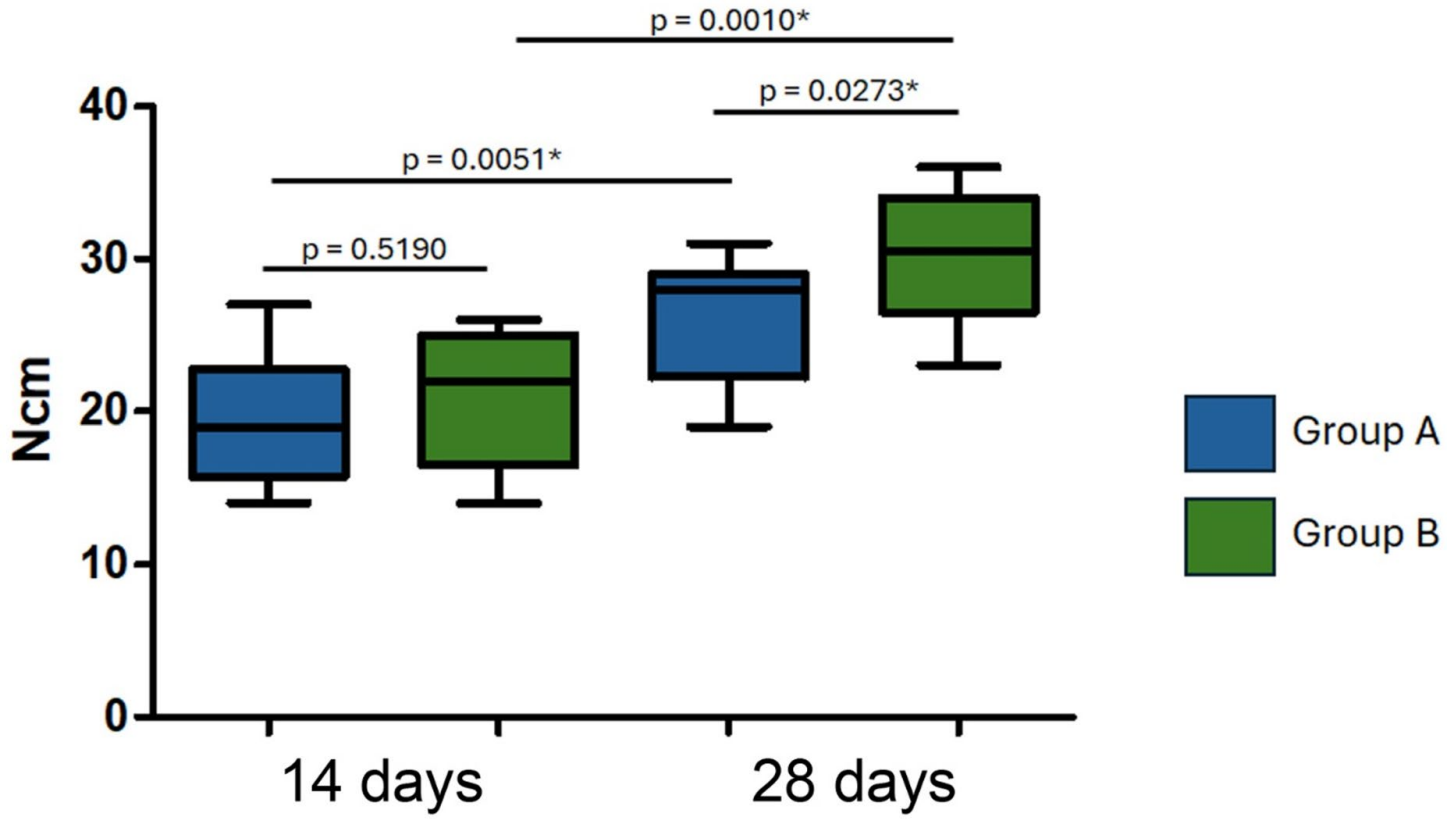
Bar graph showing mean removal torque values (in Ncm), standard deviations, and statistical analysis within and between groups at 14 and 28 days. Group A = implants with sandblasted titanium oxide surface; Group B = implants with sandblasted + hydrochloric acid-etched (35%) surface (Superiore surface). *Statistically significant difference.

In the 14-day period, the implant removal torque values ​​did not show a statistically significant difference between groups A and B, suggesting that, in this initial period, the behavior of the implants was similar in both groups. However, when analyzing the 28-day data, a significant difference was observed between the groups, with group B presenting higher removal torque values ​​(30.1 ± 4.18 Ncm) compared to group A (25.6 ± 3.95 Ncm). Furthermore, statistical analysis revealed significant differences within the groups when comparing the evaluation times (14 and 28 days). Both group A and group B showed a significant increase in the removal torque value between 14 and 28 days, indicating that the osseointegration process of the implants continues to evolve over time, resulting in greater stability after 28 days. Figure 9 shows the graph with the mean removal torque values, standard deviation and statistical analysis within and between groups.

**Fig. 10:**
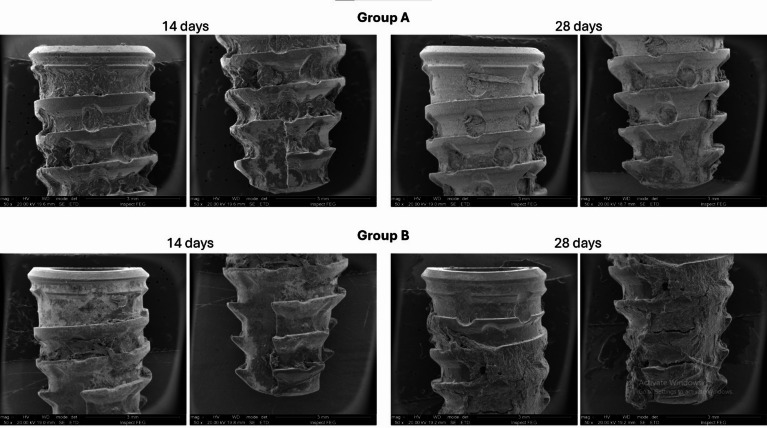
Scanning electron microscopy (SEM) images of dental implants retrieved after removal torque testing at 14 and 28 days for both groups. Group A (upper row) implants were treated by sandblasting with titanium oxide microparticles. Group B (lower row) implants were sandblasted with titanium oxide microparticles and subsequently etched with 35% hydrochloric acid (Superiore surface). The images illustrate bone tissue residues adhering to the implant surfaces, with Group B showing more extensive bone coverage over time.

Furthermore, Fig. 10 presents images obtained in SEM of the implants removed in counter torque, where it is possible to see that in the 14-day samples the amount of residual bone in both groups is very similar. However, in the samples after 28 days, the implants in group B presented a greater amount of bone tissue on the surface.

### Histomorphometric analysis

**Fig. 11:**
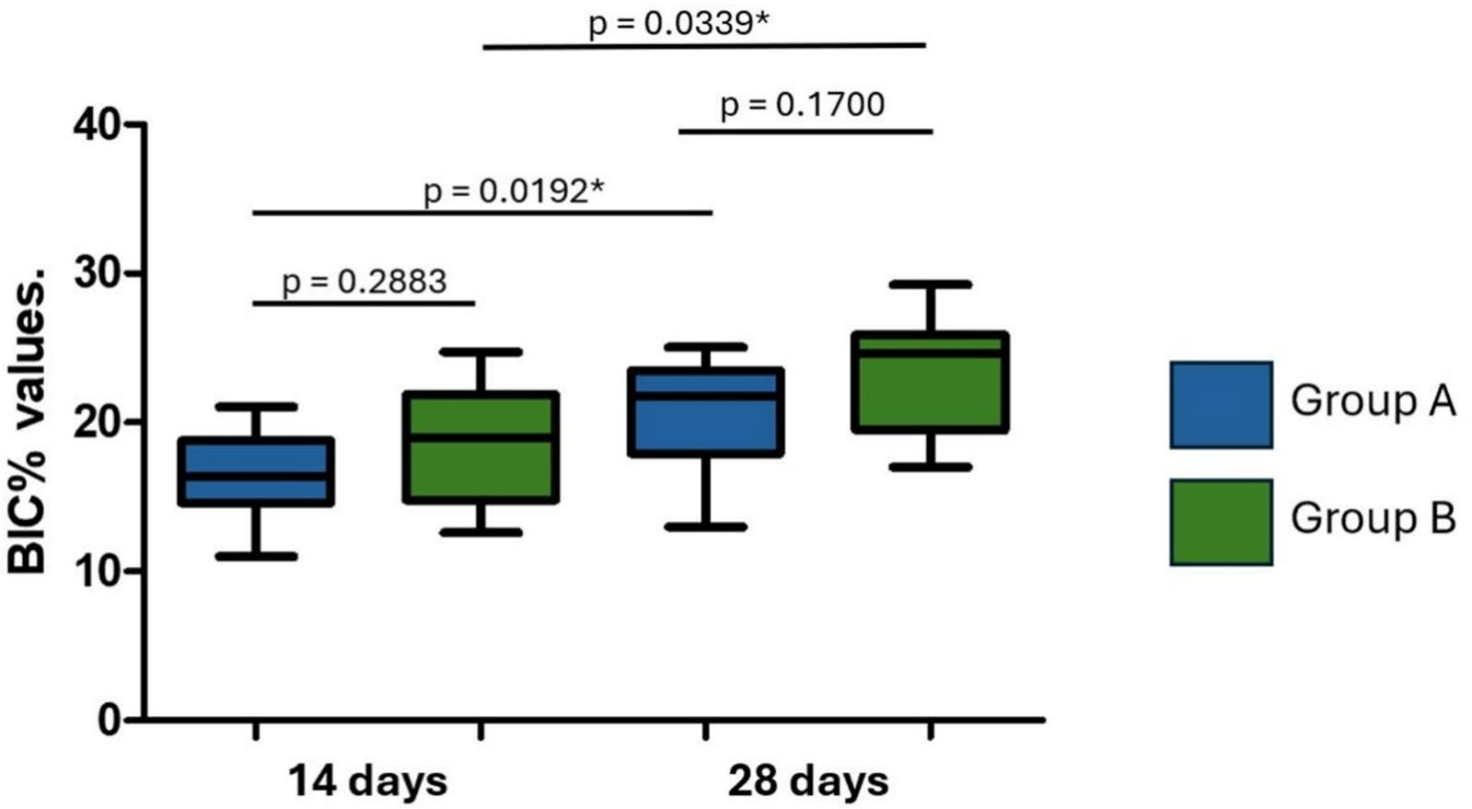
Box plots showing the distribution of bone-to-implant contact percentage (BIC%) values at 14 and 28 days for Groups A and B. Group A implants were treated by sandblasting with titanium oxide microparticles. Group B implants were sandblasted with titanium oxide microparticles and subsequently etched with 35% hydrochloric acid (Superiore surface). *Statistically significant difference.

The BIC% values ​​measured for group A were 16.4 ± 3.09% on 14 days and 20.7 ± 3.78% on 28 days. In group B, the values ​​were 18.5 ± 4.00% (14 days) and 23.3 ± 3.92% (28 days). A statistically significant difference was observed within the groups when comparing the evaluation times (14 and 28 days). However, between the groups at the same evaluation time, there were no statistically significant differences, as demonstrated in Fig. 11.

**Fig. 12:**
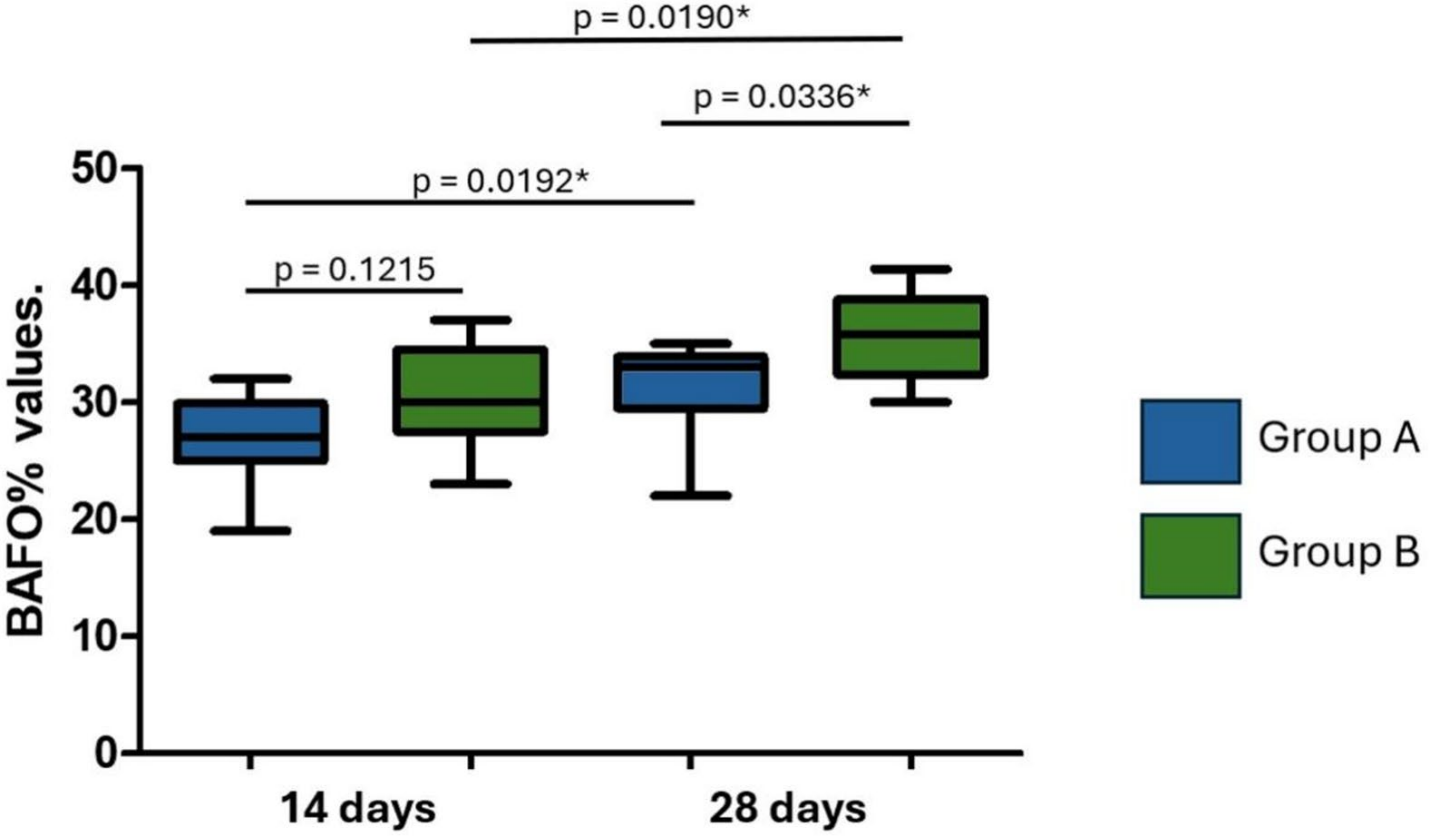
Box plots show the distribution of bone area fraction occupancy percentage (BAFO%) values at 14 and 28 days for Groups A and B. Group A implants were treated by sandblasting with titanium oxide microparticles. Group B implants were sandblasted with titanium oxide microparticles and subsequently etched with 35% hydrochloric acid (Superiore surface). *Statistically significant difference.

Regarding the BAFO% values, in group A, the measured values ​​were 26.9 ± 3.86% (14 days) and 31.2 ± 3.98% (28 days). In group B, the values ​​were 30.3 ± 4.42% (14 days) and 35.6 ± 3.78% (28 days). As with the BIC% values, statistically significant differences were observed within the groups when comparing the evaluation times. Between the groups, at 14 days, no statistically significant differences were found, but after 28 days, a significant difference was observed. Figure 12 shows the distribution of the values ​​obtained and the statistical analysis.

### Histological observations

**Fig. 13:**
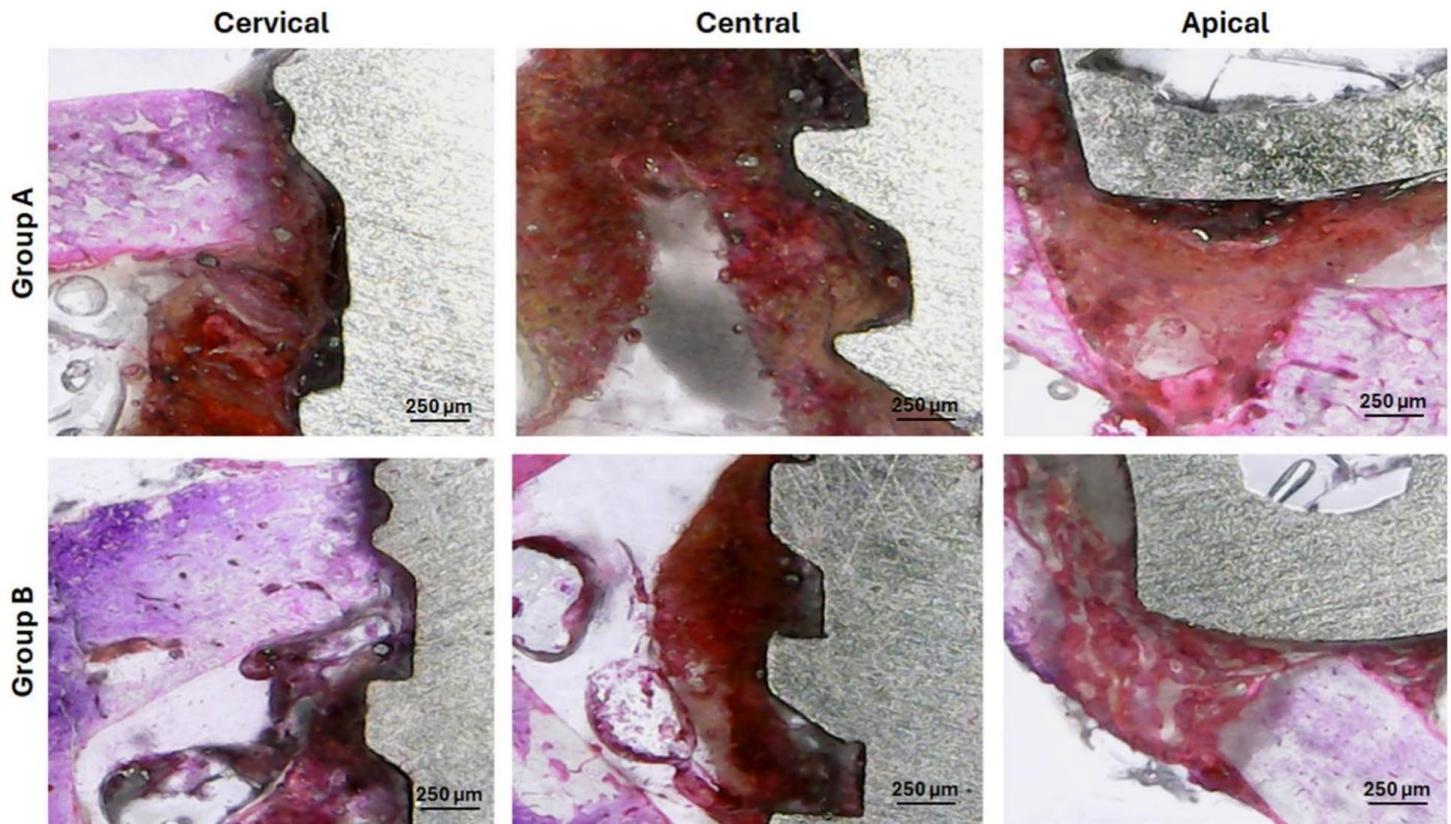
Representative images of histological sections at three regions (cervical, central, and apical) for both groups. These images correspond to samples collected 14 days after implantation. Group A (upper row) and Group B (lower row).

**Fig. 14:**
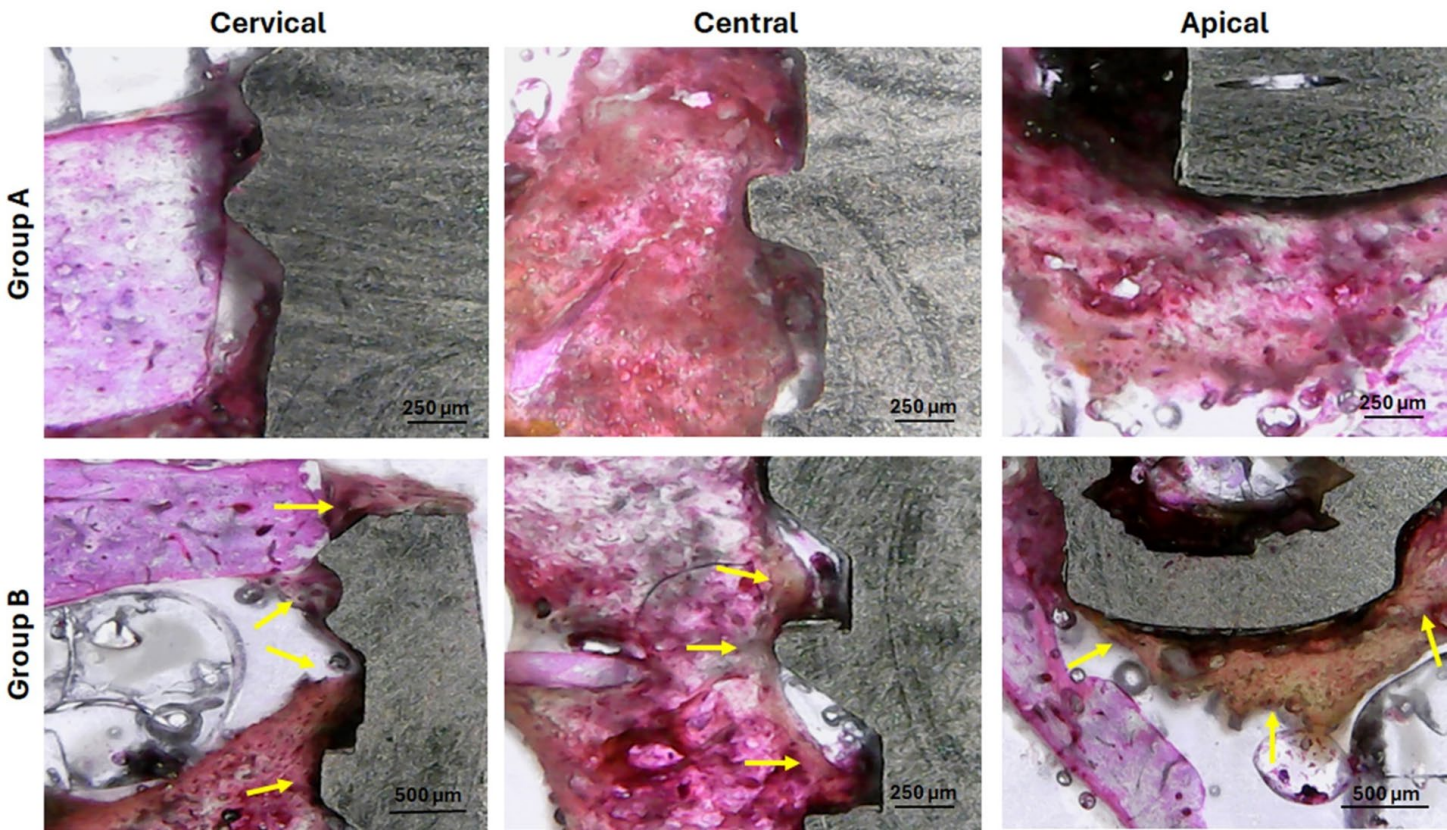
Representative images of histological sections at three regions (cervical, central, and apical) for both groups. These images correspond to samples collected 28 days after implantation. Group A (upper row) and Group B (lower row). The yellow arrows show the new bone formed in the samples of group B with a better organization compared to group A.

Analyzing the histological images, 14 days after implantations, at three different points of each sample (cervical, central and apical), we can see few differences between the groups, as demonstrated in Fig. 13. However, after 28 days, it was possible to observe better bone formation in the samples from group B compared to the samples from group A, as demonstrated in the images in Fig. 14.

## Discussion

The results obtained in the present study provide data on the biomechanical behavior of dental implants installed in an animal model with induced osteoporosis. All implants used had macrogeometry with healing chambers, which, in other previous studies, have been shown to benefit the osseointegration process compared to implants with conventional macrogeometries^10,11^. Thus, to further aid osseointegration in these types of condition (osteoporotic condition), two distinct surface treatments were compared, a conventional sandblasting treatment and a recently developed sandblasting plus acid etching^13^. Since osteoporosis is prevalent in the elderly, particularly in postmenopausal women, understanding the implications of osteoporotic conditions on implant stability and osseointegration is essential. Our results emphasize the importance of surface treatments and macrogeometry of implants in promoting osseointegration, particularly in compromised bone conditions such as osteoporosis, corroborating results presented by other authors^19,20^. Additionally, the successful induction of osteoporosis was confirmed through radiographic analysis, which showed a significant reduction in mineralized bone area in osteoporotic animals compared to healthy controls. This objective validation strengthens the reliability of the animal model and provides a solid foundation for interpreting the biological responses observed in subsequent analyses, in line with methodologies employed in previous studies using similar 2D images to validate osteoporotic conditions in animal models^21^.

### Implant stability and resonance frequency analysis (RFA)

In evaluating implant stability through resonance frequency analysis (RFA), the results revealed a significant difference between the two groups at 28 days post-implantation (E3), with Group B showing superior stability compared to Group A. This finding suggests that the surface conditioning with hydrochloric acid (HCl) in Group B may play a crucial role in enhancing the stability of implants in osteoporotic bone. The improvement in stability observed in both groups over time is in line with previous research^10,11,13,22^, which suggests that implant stability increases as the osseointegration process progresses. However, the pronounced difference at 28 days points to the importance of surface modifications in promoting more efficient osseointegration in compromised bone conditions, in agreement with other recently published studies^20,23^.

### Implant removal torque values

Regarding the removal torque values ​​(RTv) obtained in this study, these were much lower than the values ​​obtained in other studies that used healthy animals^10,13^. However, the maximum RTv provide further evidence of the enhanced osseointegration process in Group B. Although no significant difference was observed at 14 days, a significant increase in RTv was seen in Group B at 28 days, with values higher than those in Group A. This supports the idea that surface treatments involving acid conditioning contribute to better bonding between the implant surface and the surrounding bone^24^. Moreover, the increase in removal torque for both groups over time underscores the progressive nature of osseointegration, which becomes more pronounced as bone healing continues^25^.

Furthermore, the images obtained by scanning electron microscopy (SEM) showed that, after 28 days, group B presented a greater quantity of bone tissue on the implant surface, corroborating the removal torque results, which indicate better osseointegration in this group. This reinforces the hypothesis that the design or surface of the implants in group B may have provided a more favorable environment for bone formation, which in turn resulted in greater mechanical stability^10^.

### Histomorphometric analysis: bone-implant contact (BIC%) and bone area fraction occupancy (BAFO%)

The histomorphometric analysis of bone-to-implant contact (BIC%) and bone area fraction occupancy (BAFO%) revealed differences between the two groups over time. Although no statistically significant differences in BIC% were observed between groups at the same time points, Group B consistently showed higher mean BIC% values at both 14 and 28 days. This trend may suggest a surface-related influence on bone apposition over time. However, it is important to emphasize that these results reflect trends within an animal model and should not be extrapolated to clinical performance without caution.

The increase in BAFO% observed at 28 days in Group B was statistically significant and may indicate more advanced bone maturation within the healing chamber. While healing chambers are primarily designed to promote space for bone ingrowth, the surface treatment may have played a secondary role in accelerating this process, like results reported by other authors^26^.

The lack of significant differences between groups at 14 days in terms of both BIC% and BAFO% suggests that the early stage of osseointegration may not be strongly influenced by surface treatment, but rather by factors such as the initial mechanical interlocking of the implant in the bone, corroborating previous reports^27^. However, the more pronounced differences at 28 days highlight the potential benefits of surface treatments in promoting long-term osseointegration, particularly in osteoporotic bone, where bone density and quality are compromised^28^.

Although both BIC% and BAFO% are relevant metrics for evaluating osseointegration, the significant difference observed in BAFO%, but not in BIC%, at 28 days may reflect the distinct biological aspects that each parameter measures. While BIC% quantifies the direct linear contact between bone and implant surface, BAFO% assesses the total area of new formed tissues within the implant threads^29^. Thus, the higher BAFO% in Group B suggests a more advanced degree of bone infill and maturation within the peri-implant space, even though this did not yet result in a statistically significant increase in linear bone contact (BIC%). This finding may indicate that the surface treatment used in Group B promotes earlier or more substantial bone tissue formation around the implant, which could precede or eventually lead to increased BIC in longer healing periods.

From a biological standpoint, surface topography plays a critical role in modulating cellular behavior at the bone-implant interface. Micro- and nanotopographical features created by acid etching increase the surface area and favor protein adsorption, which in turn enhances osteoblast adhesion, spreading, and differentiation^20^. These processes are fundamental to initiating and sustaining osseointegration. Furthermore, surface energy changes and the possible exposure of calcium phosphate residues may further stimulate osteogenic activity. Importantly, our previous in vitro study on this specific acid-etched surface (Superiore^®^) showed enhanced osteoblast viability and adhesion, along with minimal residual contaminants, supporting its potential bioactivity and cleanliness^30^. These cellular responses may be particularly advantageous in osteoporotic bone, where the regenerative capacity is impaired.

### Study relevance

The results of this study suggest that implants with healing chambers combined with an additional acid-etching surface treatment (Group B) may offer advantages in osseointegration in osteoporotic bone within the context of an animal model. However, it is important to stress that these findings are limited to the experimental design employed and reflect biological responses in a controlled in vivo rabbit model with induced osteoporosis.

Although such surface modifications may enhance bone response under these conditions, further preclinical studies—including those with longer follow-up periods or alternative animal models—are necessary before considering potential translation to clinical practice. Additionally, while the discussion references possible benefits in patients with osteoporosis, these implications remain speculative in the absence of clinical trials.

Therefore, the conclusions of this study are limited to the animal model used, and while they may inform future hypotheses, any clinical relevance must be supported by human data.

### Limitations and future research

While this study provides valuable insights, it is important to acknowledge its limitations. The animal model, although commonly used in implant studies^14,15^, does not perfectly replicate the complexities of human bone and physiology. Further studies in human clinical trials are needed to confirm the efficacy of these surface treatments in the context of human osteoporosis. Additionally, the long-term effects of these treatments on implant stability, particularly beyond the 28-day observation period, warrant further investigation.

Future research could explore other surface treatments, combinations of biomaterials, or adjunct therapies such as bone morphogenetic proteins (BMPs) or bisphosphonates to further enhance osseointegration in osteoporotic bone. Moreover, investigating the molecular mechanisms underlying the enhanced osseointegration observed in Group B could provide deeper insights into the biological processes at play.

## Conclusion

In conclusion, the results of this study suggest that titanium implants with healing chambers and surface treatments involving titanium oxide blasting and hydrochloric acid conditioning show promising potential for improving stability and osseointegration in osteoporotic bone. Group B, with its modified surface treatment, demonstrated superior implant stability and enhanced osseointegration compared to Group A, highlighting the importance of surface modifications in overcoming the challenges posed by osteoporosis. These findings contribute to the growing body of evidence supporting the use of advanced implant technologies to improve clinical outcomes in patients with compromised bone conditions, such as the elderly.

## Data Availability

All data generated or analyzed during this study are included in this published article.
